# Supplementary figures and images for: Postural stability of 5-year-old girls and boys with different body heights
Source: PLoS One. 2019 Dec 30;14(12):e0227119. doi: 10.1371/journal.pone.0227119 (PMC6936832; doi:10.1371/journal.pone.0227119)

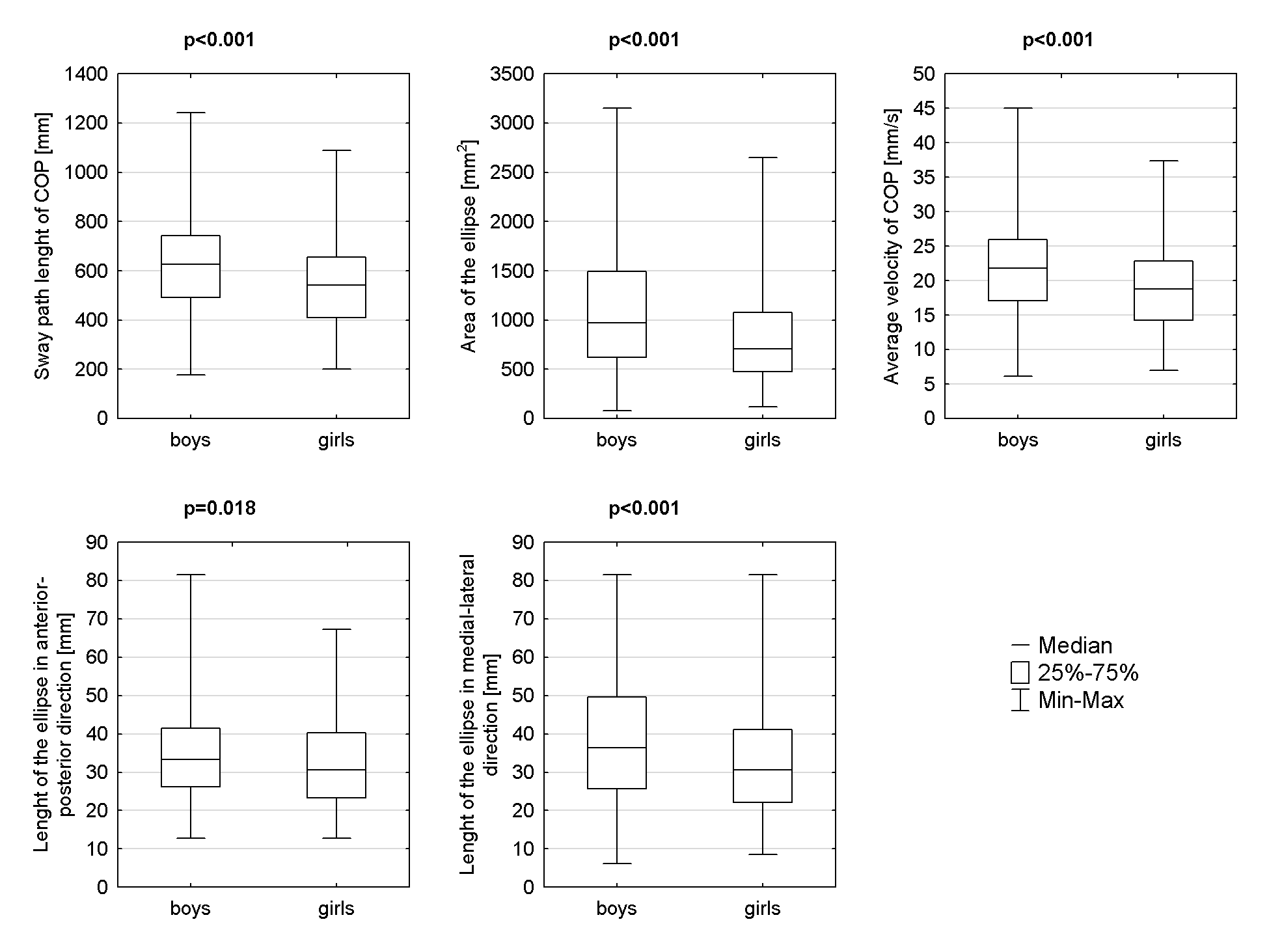

Supplement: S1 Fig — (TIF) [file pone.0227119.s001.tif]
