# Supplementary material for: Postural stability of 5-year-old girls and boys with different body heights
Source: PLoS One. 2019 Dec 30;14(12):e0227119. doi: 10.1371/journal.pone.0227119 (PMC6936832; doi:10.1371/journal.pone.0227119)
Supplement: Attachment — Submitted filename: Response do Reviewers.docx [file pone.0227119.s003.docx]

Thomas A Stoffregen, PhD
Academic Editor
PLOS ONE

We would like to thank for the review and comments regarding our manuscript titled “*Postural stability of 5-year-old children with different levels of body height*” written by Magdalena Plandowska, Małgorzata Lichota and Krystyna Górniak.

Thank you for a detailed analysis of our work and for all the comments which will undoubtedly lead to increasing the value of this article.

The changes suggested by the Reviewers have been enlisted below. In order to facilitate the assessment of the changes made in the revised version of the manuscript, they have been highlighted in grey. We hope that the changes will be satisfactory for the Reviewers and will make the paper methodologically correct and interesting for the readers.

**Response to Reviewer # 1**

Major issues

1. Regarding the comment: *There is over one page regarding basic information about the COP and how to obtain it*.

I agree that the Introduction section contains too much basic information about the COP. As suggested, this part has been reduced (lines 79-90).

2. Regarding the comment: *You are using very old references*.

Thank you for information about publications. References have been changed.

3. Regarding the comment: *You are using the term “levels” for postural stability and for body height. I would rather suggest using greater/worse/more/less/higher/lower instead of levels since there are no established levels of either*.

Thank you for your comment regarding the term “levels” for postural stability and for body height. I agree that the there are no established levels of either. I have taken them into account. According to the reviewer’s suggestion, the term “levels” has been removed. The title has been reedited. Instead of the term “levels”, I decided to use better/worse postural stability.

4. Regarding the comment: *“parents written consent” is not usually listed as inclusion criteria*.

Thank you for your comment. We have taken it into account. The Participants section has been reedited (lines 130-137).

5. Regarding the comment: *There are several language mistakes in the paper*.

Thank you for your comment. As suggested, the article has been edited by a native speaker.

6. Regarding the comment: *Can you justify the reason why you use the variables of height and width of the ellipse?*

The reason is the limitations of the measuring platform. The equipment does not provide detailed values of COP velocity in anterior-posterior (AP) and medial-lateral (ML) directions. Therefore, in order to analyse the COP in the anterior-posterior and medial-lateral direction, I used length of ellipse in the anterior-posterior direction (LoEAP, height of the ellipse) and length of ellipse in the medial-lateral direction (LoEML, width of the ellipse). I think that the selected parameters properly describe shifts of the COP and the surface of the ellipse (lines 173-186).

7. Regarding the comment: *What you describe as a qualitative assessment is wrong. Have you based your ranges on any previously published paper? If not, what is the reasoning behind allocating certain ranges to low, average, high postural stability?*

Thank you for your comments. A big number of study participants (435) made it possible to determine the levels of postural stability on the basis of the available methods of descriptive statistics. I thought that the selected criteria of the level of postural stability may constitute a reference to the assessment of postural stability in children from this age group. But I have not based ranges on any previously published paper. I decided to remove a qualitative assessment. Therefore, the section Stance analysis (lines 173-186) and the section Results were reedited (lines 215-240).

8. Regarding the comment: *Can you justify the statistical analysis used for your variables? Why did you use Chi-Square statistics?*

The Shapiro-Wilk test was used to analyze whether the variables had a normal distribution. Data normality was rejected, so the Mann-Whitney U test was used to examine differences between girls and boys.

The Chi square test was used to identify significant differences in the percentage of children with different postural stability. I decided to remove qualitative assessment, so the Chi square test was removed, too.

9. Regarding the comment: *Your discussion includes a lot of information from different papers and researchers but little from your own study. You are not addressing the results from each of your five parameters and how each of them are relevant to postural control.*
Thank you for your comments. The section Discussion has been reedited.

Minor issues

1. Regarding the comment: *By this sentence it looks like you say that adult levels of postural control are attained at 6 years of age. You should speficy more or rephrase.*

The reviewer`s suggestion has been taken into account in the manuscript. This sentence has been corrected (lines 105-106).

2. Regarding the comment: *Energy is not a physical ability, please rephrase.*

It has been corrected (line 120-121).

3. Regarding the comment: *“mount of sway” not sways.*

Thank you for your comment. It has been corrected according to the suggestion (line 125).

4. Regarding the comment: *In your Initial medical check-ups you assess general health as an exclusion criteria and that does not appear listed in the exclusion criteria previously mentioned. Please clarify whether this was or not another exclusion criteria.*

Thank you for this comment. Bad general health was another exclusion criterion (lines 132-134).

5. Regarding the comment: *The sentence is confusing, please check language grammar.*

Thank you for this comment. This sentence has been reedited (166-168).

6. Regarding the comment: *You mention three sway parameters and then you list 5: COP area, ellipse area, COP path, COP velocity, height of ellipse, width of ellipse.*

Thank you for this comment. A technical error.

7. Regarding the comment: *Review the bibliography. There are some mistakes.*

Thank you for your comment. The bibliography has been corrected.

**Response to Reviewer #2:**

Thank you for reading our article. According to the comments of the reviewers, the manuscript has been corrected.
