# Supplementary material for: Postural stability of 5-year-old girls and boys with different body heights
Source: PLoS One. 2019 Dec 30;14(12):e0227119. doi: 10.1371/journal.pone.0227119 (PMC6936832; doi:10.1371/journal.pone.0227119)
Supplement: Attachment — Submitted filename: Response do Reviewers.docx [file pone.0227119.s004.docx]

Thomas A Stoffregen, PhD
Academic Editor
PLOS ONE

We would like to thank for the review and comments regarding our manuscript titled “*Postural stability of 5-year-old girls and boys with different body heights*” written by Magdalena Plandowska, Małgorzata Lichota and Krystyna Górniak.

Thank you for a detailed analysis of our work and for all the comments which will undoubtedly lead to increasing the value of this article.

The changes suggested by the Reviewer 3 have been enlisted below. In order to facilitate the assessment of the changes made in the revised version of the manuscript, they have been highlighted in grey. We hope that the changes will be satisfactory for the Reviewer and will make the paper methodologically correct and interesting for the readers.

**Response to Reviewer # 1**

Thank you for a detailed analysis of our work and for all the comments which undoubtedly leaded to increasing the value of this article.

**Response to Reviewer # 3**

1. Regarding the comment:*“Gender” is utilized throughout the paper, but the proper term for what was studied is “sex.”*
Thank you for your comment. We have taken it into account.

2. Regarding the comment: *Page 7, line 167 - Other experiments of postural sway have utilized marked stance widths. Were children told to stand “normally”? Later (page 12, line 272) states that girls had a smaller stance….*
Thank you for your comment. The sentence (page 12,line 279-281) has been corrected.

3. Regard the comment: *Page 9, line 197* - *The phrase “statistically significant” indicates that some form of analysis was used, but this doesn’t say what analysis. Mann-Whitney U is stated later, but proper reporting is (U = xxx, p=0.02).*
Thank you for your comment. It has been corrected according to the suggestion.

4. Regard the comment: *Page 10, line 220 - This also needs to proper statistical reporting (U=xxx, p=.xxx). This is seen throughout the rest of the manuscript.*
Thank you for your comment. We have taken it into account.

5. Regard the comment: *Page 14, line 312 - This paragraph seems like it should be in the into, not the discussion.*
Thank you for your comment. This paragraph has been included in the Introduction (page 5-6, line 118-121).

6. Regard the comment: *Page 14, line 315* - *I am confused on why this paragraph opens up with “environment and socio-cultural factors,” and then presents no discussion of these factors. What is discussed in this section seems like it would fit in the introduction as motivation. A discussion of said environment and socio-cultural factors would be appropriate here, and would help provide insight into the differences being seen.*

Thank you for your comment. As suggested, this paragraph has been reduced (page 14, line 317-334) and part of it has been included in the Introduction (page 6, line 116-127).

Yours sincerely,

Magdalena Plandowska

Jozef Pilsudski University of Physical Education in Warsaw,

Faculty of Physical Education and Health, Biala Podlaska, Poland
